# The Peer-Reviewed Literature on Undergraduate Education for Public Health in the United States, 2004–2014

**DOI:** 10.3389/fpubh.2014.00223

**Published:** 2014-11-17

**Authors:** Connie J. Evashwick, Donghua Tao, Lauren D. Arnold

**Affiliations:** ^1^College of Health and Human Services, George Mason University, Fairfax, VA, USA; ^2^Medical Center Library, Saint Louis University, St. Louis, MO, USA; ^3^College for Public Health & Social Justice, Saint Louis University, St. Louis, MO, USA

**Keywords:** public health pedagogy, undergraduate liberal arts education in public health, undergraduate education for public health, public health workforce education in the U.S., literature review of education for public health, peer-reviewed literature on undergraduate education for public health

## Abstract

The education of undergraduate college students in the field of public health has burgeoned over the past decade. Professional literature in peer-reviewed journals is one indicator of the status of a field of study and its related body of knowledge. It is also a mechanism for sharing information among professionals about challenges, issues, experiences, and best practices. The purpose of the literature review conducted here was to describe the status of the peer-reviewed literature over the past decade pertaining to the education of undergraduates about the field of public health in the United States (U.S.). A literature search was conducted of three databases: PubMed, Scopus, and ERIC. Inclusion criteria were publication date from January 1, 2004 through July 31, 2014; written in the English language; pertaining to undergraduate education in the U.S.; and a focus on public health as the primary discipline. Public health was searched as an overarching discipline; articles focused on sub-disciplines or other health professions disciplines were excluded. The search resulted in 158 articles. Each of the authors reviewed the abstracts for all articles and read full articles when necessary. The result was 23 articles that were then considered in depth. The articles were categorized according to their primary theme: curriculum, courses, learning objectives (*N* = 14); evaluation of teaching method (*N* = 3); case study (*N* = 3); career path and advising (*N* = 2); accreditation (*N* = 1). Year of publication and journal were also examined. The results of the literature search lead to several observations about how the peer-reviewed literature has been used to date and how it could be used to advance the emerging field of undergraduate education for public health.

Peer-reviewed literature is one indicator of the status of a field of study (1). It provides historical perspective on the evolution of theory and empirical evidence, shows the most recent threads of investigation, and showcases experts in the field. For those seeking an introduction to a field, a search of the peer-reviewed literature also provides a means to identify key themes and find best practices. The purpose of this study was to examine the peer-reviewed literature pertaining to undergraduate education for public health (UGPH) in the United States (U.S.).

Throughout this paper, the acronym UGPH is used to refer to the education of undergraduate college students *about the field of public health*, such as majors or minors in public health or courses in epidemiology or environmental health. This is not to be confused with education *about public health* intended to influence the behavior of college students, such as stop smoking or safe sex campaigns.

## Background

A brief summary of the forces driving UGPH provides a context by which to examine the literature. UGPH programs have been offered for decades, with many based in universities having schools of public health. However, in 2003, the Institute of Medicine (IOM) recommended that all U.S. undergraduates have basic education in public health (2). Since then, the number of institutions offering UGPH majors and minors has grown dramatically, including many in community colleges, liberal arts colleges, and universities without schools or programs of public health (3).

Following the IOM report, four national associations joined together to develop the concept of “the educated citizen” as one who is knowledgeable about public health. They created the learning outcomes model, based on the liberal education and America’s promise (LEAP) framework promoted by the Association of American Colleges and Universities (AAC&U), appropriate for all undergraduate students regardless of major or minor (4).

Association of American Colleges and Universities, working with the Association for Prevention Teaching and Research (APTR), developed the first detailed set of recommendations for UGPH. These included a sample curriculum, templates for three introductory courses, and a compilation of 15 case studies from undergraduate public health programs (5). About this time, leading publishers began to offer textbooks on public health topics aimed at undergraduates (6).

In 2012, the Association of Schools and Programs of Public Health (ASPPH) convened an expert panel to develop the critical component elements (CCEs) of an Undergraduate Major in Public Health. The CCEs describe basic, essential elements for an undergraduate major in public health while allowing considerable flexibility on how that major will be administered (7).

The Public Health Accreditation Board was launched in 2011, offering accreditation for the first time at a national level to local and state public health departments (8). Standard eight pertains to ensuring the education of the public health workforce (9). The need for UGPH has also been supported by the American Public Health Association, with a 2012 report stating the potential significance of UGPH for the public health workforce (10).

In 2014, the accrediting body Council on Education for Public Health (CEPH) added the option of accreditation for free-standing baccalaureate programs in public health (11). Previously, UGPH programs had been accredited only if they were offered by a school of public health or graduate program in public health. The growing number of free-standing public health majors prompted CEPH’s action as a way to establish consistency and a baseline for quality. The CEPH accreditation standards are based on the CCEs and outline program requirements in further detail.

These aforementioned UGPH initiatives are public health-centric. The health professions fields of medicine and nursing, as well as others, have spent considerable effort examining the education of their respective disciplines, including the relevancy of public health (12). Recently, “population heath” and “global health” have each received increasing attention as essential elements of the healthcare system, and thus, of healthcare professionals’ education (13, 14).

A literature review by Evashwick et al. concluded that there is indeed concern within the profession about the content and quality of education for the public health workforce, but a variety of barriers prevent literature on the pedagogy of public health training from being published or searched (15). The literature review identified 464 articles published between 2000 and 2012, with 6 focused on UGPH.

This evolution in UGPH begs the question: has the growth in undergraduate academic-based programs been accompanied by discussions in the peer-reviewed literature among the engaged faculty and practitioners?

## Objective

The objective of this project was to describe the status of the peer-reviewed literature pertaining to UGPH in the U.S. over the past decade. As undergraduate educational opportunities have developed, questions have been raised by the academic and the practitioner communities about content, quality, workplace relevancy, and career progression, as well as relationship to subsequent graduate education (16) and the value of accreditation. The cohesion or disciplinary structure of the field is also in question, reflecting the debate about whether public health is a free-standing profession or a subset of another discipline (17). All of these questions have implications for academic programs at the undergraduate level. We looked to the published peer-reviewed literature for guidance about these issues.

## Methodology

A literature search was conducted of three comprehensive bibliographic databases: PubMed, Scopus, and ERIC. Collectively, these three databases were expected to capture the majority of articles pertaining to the pedagogy of UGPH. Two separate search activities were performed in each database. The first search used public health as a specific discipline, and the second search was of sub-topics within the discipline, such as environmental health, healthcare administration, nutrition, health promotion, and global health. The search terms included Public health/education; Education, Public Health Professionals; undergraduate; bachelor degree; baccalaureate degree; Environmental Health; Health Services Administration; Nutritional Sciences; Health Promotion; and Global Health.

The field of public health in the U.S., as well as in other countries, has various sub-divisions, some of which are formally recognized by the accrediting bodies, and some of which have grown up separately with parallel accreditations for academic programs. From the academic perspective, a profession that has distinct licensing or certification is likely to have an accompanying pedagogy that reflects the unique aspects of that field. Many of these sub-disciplines also have distinct undergraduate education majors in U.S. colleges and universities. Thus, in conducting the literature search, those sub-fields that have their own accreditation were considered specialties within public health. The search was constructed to find articles that emphasized the general field of public health. Although the search included the sub-disciplines, articles emphasizing *only* the sub-discipline were excluded from the search.

The “gray literature” was not combed. Examining the citations of articles published in peer-reviewed journals turned up reports, monographs, conference presentations, policy statements, and other potentially relevant material. However, in the absence of an intellectual framework or pragmatic infrastructure for searching the unpublished information in a comprehensive, systematic way, it was determined not to include gray literature in the study.

The initial search results were narrowed down to the articles that had abstracts, were published between January 1, 2004 and July 31, 2014, and written in the English language. This yielded a total of 158 articles. Each of the three authors reviewed all abstracts according to pre-determined criteria, reading the full article when the abstract was not sufficient to determine if the focus was UGPH education. Articles were eliminated for any of the following reasons:

(1) They focused on a country other than the U.S. “Written in the English language” was used as a proxy for the U.S. as a preferable search term, but yielded articles from Britain, Australia, New Zealand, and other English-speaking countries. These were then eliminated, as their educational systems are different.
(2) The focus was “undergraduate medical education.” In the U.S. model, undergraduate medical education is post-baccalaureate and is formally graduate-level education.
(3) Articles pertaining to the education of nurses and other health professionals, whether at the graduate or undergraduate level, were eliminated because they considered how to incorporate public health topics into existing curricula for a different health profession, rather than how to specifically shape an undergraduate curriculum with a focus on public health.
(4) Public health and its sub-topics were only one component of or one course for other non-health undergraduate degree programs. For example, an article about teaching statistics to undergraduates met the initial criteria and appeared in the search. However, the article incorporated an example from public health but was about techniques for teaching statistics in general, not just for those students studying public health.

The total number of articles in each of the above categories is not presented, as many articles fell into more than one category. After reaching 100% agreement among all three authors, a total of 23 articles remained for review. These are displayed in the Supplementary Material.

## Findings

The articles were examined for their characteristics and content. The 23 articles fell into five broad categories: learning outcomes and curriculum; teaching methods; case studies of specific university programs; career paths and advising; and accreditation (Table 1). The majority were about the rationale and goals for undergraduate education in public health and recommendations for the corresponding curriculum, as well as descriptions of specific courses.

**Table 1:** **Content focus of articles**.

Focus	*N* (%)
Learning outcomes and curriculum	14 (61%)
Teaching methods	3 (13%)
Case studies	3 (13%)
Career paths and advising	2 (9%)
Accreditation	1 (4%)
Total	23

Between January 1, 2004 and July 31, 2014, some years had no articles published in peer-reviewed literature (Table 2). In 2008, 11 of the 23 articles appeared. This was in part because one journal had a supplement on UGPH.

**Table 2:** **Number of articles published by year**.

Publication year	Number of articles
2004	1
2005	0
2006	0
2007	0
2008	11
2009	0
2010	3
2011	3
2012	2
2013	3
2014	0

During the 2004–2014 timeframe, articles on UGPH have appeared in seven journals (Table 3). Two journals published 65% (*n* = 15) of the articles identified in the search.

**Table 3:** **Number of articles by journal**.

Journal	Number of articles
American Journal of Preventive Medicine	9
Public Health Reports	6
Education and Health (Abbington)	2
Journal of Public Health Management and Practice	2
Academic Medicine	1
Health Education Behavior	1
American Journal of Public Health	1
American Journal of Health Education	1

The number of individual authors was 44. Several authors had authored or co-authored multiple articles.

## Discussion

The peer-reviewed literature is one means of communicating key issues pertaining to an emerging or changing field, such as UGPH. During more than a decade following the IOM recommendation to educate all undergraduates about public health, very few articles have been published to indicate that those working in the field have been exchanging information through the peer-reviewed literature about how to implement this recommendation. The paucity of literature about pedagogy in public health journals is noteworthy, as it is the public health faculty who has the subject matter knowledge to train the future public health workforce. Of all the faculty engaged in teaching undergraduates about public health, whether as a major/minor or as an educated citizen, a small number over the past decade have written for the professional literature indexed in PubMed, Scopus, or ERIC.

It should be noted that the peer-reviewed literature about UGPH in the U.S. is not the only indication of attention to pedagogy. Other sources of information include education journals, literature published by and about the teaching of public health in other countries, literature about the pedagogy of sub-disciplines of public health and of other health professions disciplines that incorporate public health into their curricula, and the gray literature. Additionally, ideas and experiences are shared through websites, blogs, listservs, and email exchanges. Conferences, accreditation preparation and site visits, and other in-person mechanisms are yet other means of spreading information. Nonetheless, the peer-reviewed literature can be a powerful way to present data, spark controversy, and share ideas, particularly for those who are new to the field and are searching for an introduction.

The results of the literature search lead to the following observations:

- Health professions disciplines, particularly medicine and nursing, have given extensive thought and practical evaluation to the incorporation of public health content within their curricula.
- Some sub-disciplines of public health, including environmental health, health education, nutrition, and health management and policy, have many more articles in the peer-reviewed literature on pedagogy than the general field of public health. Health administration is just one example of a facet of public health that has an entire journal devoted to its pedagogical issues, *the Journal of Health Administration Education*.
- Faculty in other countries have given considerable thought to the pedagogy of public health, including at the undergraduate level. Developing countries in particular have few graduate-level public health programs, which leads to a focus on how to train the future public health workforce at the undergraduate level.
- Some public health topics, such as tobacco control, have been examined for how to incorporate them into the education of undergraduate students in the U.S., whether in health professions disciplines or for all undergraduates. Education about public health aimed at behavior modification may offer an opportunity to expand into discussion about the broader field of public health.
- Few articles reported on rigorous evaluations of teaching methods or content pertinent to UGPH in the U.S. Of the 23 articles in the final list, only 3 were detailed assessments of teaching techniques.
- Only two articles published during the past decade related UGPH training to subsequent career choices or public health careers.
- Unlike other disciplines in the health professions, no journal has historically focused on pedagogy for training public health professionals.

The number of colleges and universities in the U.S. that currently offer public health as a major, minor, or elective is growing. Universities with schools of public health and other health professions disciplines might be able to tap experts to teach, but small liberal arts and community colleges might rely on faculty to teach public health who come from other disciplines and are less familiar with the content, or expert practitioners from the field who know the content but are less experienced with academics. The readily available published peer-reviewed literature can be an important place for faculty to turn to for guidance.

Efforts are underway in the U.S. to revise the curriculum guidelines for teaching public health professionals at the master’s and doctoral levels (18). As the pedagogy for these advanced levels is evaluated and revised, considering the articulation of graduate training with undergraduate training in public health will be essential. This is the case not just for public health as an independent profession, but for other health professions disciplines as well. As the educational requirements are changed for undergraduates who are seeking specialized study in medicine, nursing, dentistry, veterinary medicine, and other health professions, will the changes contribute to, complement, or compete with undergraduate education in public health? The published academic literature provides one easily accessed forum to exchange ideas about vertical and horizontal curricula articulation, challenges, and best practices.

## Conclusion

To the extent that peer-reviewed literature is one indication of the sophistication of an academic discipline, the field of UGPH is still emerging. The literature about the pedagogy for UGPH can be expected to grow over time. However, the more quickly information is shared, the more likely education will advance in content taught, teaching effectiveness, curriculum articulation, and the relevance of UGPH education to developing an educated citizenry and to preparing students for careers in public health and related health professions.

## Conflict of Interest Statement

The authors declare that the research was conducted in the absence of any commercial or financial relationships that could be construed as a potential conflict of interest.

## Supplementary Material

The Supplementary Material for this article can be found online at http://www.frontiersin.org/Journal/10.3389/fpubh.2014.00223/abstract
